# The dynamic growth of bacterial cultures: real-time Bayesian estimation of substrate uptake rates in fed-batch fermentations of *E. coli*

**DOI:** 10.1007/s00449-025-03251-0

**Published:** 2025-11-08

**Authors:** Maximiliano Ibaceta, Mark-Richard Neudert, Nuno Marques, Stefan Kahrer, Christoph Herwig, Andreas Steinboeck

**Affiliations:** 1 Bioprocess and Digitalization, Fermify GmbH, Richard-Neutra Gasse 5, 1210 Vienna, Austria; 2 Automation and Control Insitute (ACIN), TU Wien, Gußhausstraße 27-29, 1040 Vienna, Austria; 3 Lisalis GmbH, Vienna, Austria

**Keywords:** Substrate uptake dynamics, Adaptive state and parameter estimation, Bayesian estimation, Particle filter, Bioprocess engineering

## Abstract

Accurate real-time estimation of system states and metabolic parameters is essential for effective bioprocess control. However, the dynamics of microbial adaptation—the rate at which a microorganism adapts to changes in the substrate concentration—is often overlooked, leading to early-stage plant-model mismatches and inaccurate estimation of relevant parameters, such as the biomass yield on carbon source ($Y_{XC}$) or the maximum substrate uptake rate ($q_S^{\text {max}}$). This work introduces a novel model-based observer for simultaneous state and parameter estimation that explicitly accounts for substrate uptake dynamics. By defining the substrate uptake rate ($q_S$) as a state variable and introducing a random variable ($\lambda$) to represent the biomass-specific substrate uptake adaptability rate, we construct a Bayesian estimator that allows proper determination of the states and parameters in fed-batch fermentations of *E. coli* while maintaining near-zero centered residuals between the plant output and the proposed model stoichiometry. This work advances methods for robust state and adaptive parameter estimation in dynamic bioprocess environments under uncertainty.

## Introduction

Bioprocess development aims to establish a robust production strategy throughout the product life cycle. However, biological processes are often subject to disturbances arising from biological batch-to-batch variability and operator interventions that require adequate process estimation and control solutions. These compensation strategies depend on an accurate estimation of key metabolic parameters such as the biomass yield on carbon source $Y_{XC}$, the specific growth rate $\mu$, and the maximum substrate uptake rate $q_S^\text {max}$, alongside the system states [1–3]. Direct measurement of these quantities in real-time is challenging due to delays or infeasibility in sampling as well as the potentially high manual effort required [2–5].

The rising implementation of process analytical technologies (PAT) in bioprocesses engineering has enabled real-time approximation of relevant properties of microbial cultures throughout a fermentation [6–8]. Access to instantaneous yields, biomass concentration, and cell-specific rates is possible thanks to the implementation of soft-sensors [9–11] and estimators [2, 12–14]. However, the experimental validation and unsupervised implementation of these estimators remains problematic [3, 13] as the performance of these model-based observers is either dependent on the proper initialization of the state variables [15], the fidelity of the model and parameters to represent the physical plant (structural identifiability), or both. In this regard, the availability of state observers coupled with adaptive parameter estimators that can simultaneously address initialization and parameter uncertainties is limited. Table 1 summarizes the key developments on estimation applications on *E. coli* cultivations based on the Kalman Filter (KF), Asymptotic observer (AO), Extended Kalman Filter (EKF), Unscented Kalman Filter (UKF), and the Particle Filter (PF), together with the required parameter knowledge for these estimators, as well as the plant output and initialization conditions.

**Table 1:** Survey of state observers and adaptive parameter estimators on *E. coli* strains in fed-batch mode derived from the Monod model

References	Method	Estimation goal	A priori Param. knowledge	Output $\boldsymbol{y}(t)$	$t_{init}$ [h]
[16]	EKF	${\hat{q}}_{Ac}(t), {\hat{\mu }}_\text {max}^{O_2}(t)$	$\boldsymbol{\theta }$	*X*(*t*), off-gas	0
[17, 18]	EKF/AO	${\hat{q}}_{Ac}(t)$	Yields	Various	$t_\text {fed-batch}$
[19]	KF	${\hat{\mu }}_\text {max}(t)$	$Y_{XC}, k_S$	*S*(*t*)	$t_\text {late-batch}$
[20]	AO	${\hat{\mu }}(t)$	$Y_{XC},k_S$	*OUR*(*t*)	0
[22]	UKF	*X*(*t*)	$\boldsymbol{\theta }$	*Ac*(*t*)	0
[21]	UKF	${\hat{\mu }}(t)$	$\boldsymbol{\theta }$	Off-gas	$t_\text {late-batch}$
[13]	PF	${\hat{q}}_S^\text {max}, {\hat{Y}}_{XS}, q_S$	$k_S$	Off-gas	0

$\boldsymbol{\theta }$: Full knowledge of the parameter vector required

off-gas: [*CER*(*t*), *OUR*(*t*)]

Although the performance of the estimators presented in Table 1 is satisfactory in most cases, none of the aforementioned filters can simultaneously deal with uncertainty in both the states, and the parameter vector $\boldsymbol{\theta }$ without significantly deviating from the plant output reference. This is particularly evident in conditions where standard kinetic models with time-invariant parameters [16–18, 21–24] are not flexible enough to capture the dynamic nature of microbial adaptation. Examples for this dynamic include the transition from the lag phase to exponential growth, the microbial response to substrate pulses in fed-batch mode, or the behavioral change of microbial cultures at high cell densities [25–28]. Misinterpretation of these dynamics may lead to unreliable parameter and state estimates [29, 30] arising from the potential information loss [31, 32], and increased sampling efforts to adjust the system states [3, 33].

While the standard Monod model [34, 35] assumes an instantaneous response in biomass growth and substrate uptake rate in the presence of external substrate, the microbial behavior during the lag-phase and the experimental adaptation observed in substrate pulse experiments [13, 36] highlight the need to consider alternatives to model and estimate the growth of microbial cultures beyond the strategies proposed by the authors in Table 1 and the re-initialization and heuristic compensation efforts from other researchers [33, 37–40].

Reformulating time-invariant parameters as time-varying is a promising solution to address the drift between the model-estimated and experimentally measured states. This flexibility has significantly improved state estimation in bioprocess engineering [13, 14]. These time-varying parameter (TVP) estimation strategies often rely on an update policy that minimizes an error between a plant output: such as the Oxygen Uptake Rate (OUR) and/or the Carbon Evolution Rate (CER), and a model-computed response (output) such as the expected system stoichiometry from where reaction rates like $r_{O_2}$ and $r_{CO_2}$ can be obtained [3, 10].

For the computation of the model output, propagation of the system states in time through a dynamic model is required. In this regard, sequential Monte Carlo (SMC) Bayesian methods [41] have proven to be a valuable tool for the computation and propagation of the system states considering the highly nonlinear formulation of microbial fermentation models, as well as enabling adaptive parameter evolution in response to changing conditions [42, 43].

Two notable studies [13, 14] have exploited this approach in different settings. Sinner et al. (2022) implemented a particle filter estimator on continuous cultures of *Corynebacterium glutamicum*, demonstrating that metabolic capacities evolve during long-term fermentations. Their findings emphasized the need for parameter adaptation to prevent model-plant mismatch. Similarly, Müller et al. (2023) applied particle filtering to simultaneously estimate metabolic parameters ($q_S^{max}$, $Y_{XC}$) and system states in fed-batch cultures of *Escherichia coli*. Both studies underscored the challenges of validating the true values for $q_S^\text {max}$ or $\mu _\text {max}$ from the Monod model at later fermentation stages. Nevertheless, they highlighted the relevance of adaptive parameter estimation in reconciling the plant-model mismatch concerning the latent state (Biomass). A key consideration for such estimators is determining which Monod model parameters can be reformulated as time-varying, given that their sensitivity and observability may vary throughout the fermentation [29, 44–46]. While Müller et al. (2023) attempted simultaneous state and adaptive parameter estimation, their PF algorithm prematurely discarded high $q_S^\text {max}$ values, creating a significant plant-model mismatch towards the end of the lag-phase. If the standard Monod model used in both studies is unable to represent the plant behavior, even when adaptive estimation of the parameters is attempted, then the possibility of a structural problem in the Monod model must be explored.

If an upper bound for $q_S^\text {max}$ can only be estimated in the batch-phase, where substrate is abundant, it is then necessary to build a model-based observer that can accurately capture the behavior of the microbial culture in this phase and must consider the influence of lag or activation phase, as well as the existing correlation between $\mu _\text {max}$ and $Y_{XC}$ in early batch stages. Additionally, said estimator should also provide an explanation for the hypothesized $q_S^\text {max}$ decay during the substrate-limited phase in fed-batch cultures, while adapting to potential yield changes in time and providing feasible state estimates. [14, 36]. Krichen et al., 2018 [28] already provided experimental evidence regarding the relationship between decaying $q_S$ rates and increasing biomass concentrations. Still, the transition between the standard Monod-based and biomass-dependent kinetic is unclear.

These limitations in structural identifiability and requirements in model adaptability highlight the need for a systematic modeling and estimation approach that can handle both theoretical and practical challenges in bioprocess monitoring. Such an approach is crucial for reliable digital twin implementations and advanced bioprocess control [2, 47].

The goal of the present contribution is to develop a robust model-based observer for real-time bioprocess monitoring that captures microbial adaptation dynamics to effectively estimate $q_S^\text {max}$ and $Y_{XC}$, whilst providing reliable state and adaptive parameter estimates in the carbon-limited phases. To accomplish this goal, we propose reformulating the standard Monod kinetic model into a dynamic model by considering the biomass-specific substrate uptake $q_S$ as a state variable $q_S(t)$. To justify $q_S(t)$, we hypothesize that its time-scale [15, 48] is similar to the dynamics of biomass and substrate and therefore we incorporate a time-varying parameter $\lambda$ [g g$_{DCW}^{-1}$ h$^{-2}$] that accounts for the biomass-specific substrate uptake adaptability rate. Given the impossibility of systematically capturing all the contributions emerging from batch-to-batch variability and other potential triggers for microbial adaptation, $\lambda$ should also be modeled as a random variable.

We hypothesize that the adaptability rate $\lambda$ reflects a biological activation process, where changes are triggered by specific physiological or environmental conditions rather than evolving smoothly under Gaussian fluctuations. Such activation-driven dynamics can produce abrupt or asymmetric variations, thereby stressing the Gaussian-prior assumptions of KF-based methods, making the Particle Filter a more suitable Bayesian estimation framework for modeling such processes.

This new formulation should allow a flexible realization of growth rate trajectories, allowing the estimator to distinguish between acceleration and growth at $q_S^\text {max}$. This new formulation shall enable robust simultaneous state and adaptive parameter estimation, regardless of the observed variability between batches, and does not require phase-dependent model segregation methods [4, 49].

This paper is structured as follows: We first illustrate a systematic error from the nonlinear least squares parameter estimation method when online signals are incorporated into the fitting phase of the standard Monod model. To partially overcome this bias, we experimentally determine a probability density function for the parameters in the Monod model by Monte Carlo resampling over fractions of the experimental dataset. From this parameter density function $p(\boldsymbol{\theta })$, we simulate a plant with known $q_S$ adaptation dynamics and attempt to retrieve the true parameters and states using Bayesian estimation in a particle filter using either the standard Monod kinetic model and our proposed dynamic $q_S$ model for the state transition step. The resulting parameters are compared to highlight the consequences of neglecting the hypothesized microbial adaptation dynamics using the Kullback-Leibler divergence to quantify this information loss. Finally, we perform Bayesian estimation over the original dataset using the proposed model, resulting in significant improvements in joint state and parameter estimation compared to existing methods in the literature.

## Materials and methods

### Cultivation experiments

All experiments in the paper were conducted using a derivative of the BL21(DE3) strain of *Escherichia coli*, grown in DeLisa minimal medium [50] supplemented with glycerol (8 g/L) as the sole carbon source. The pre-inoculum was cultivated in DeLisa medium at 37$^{\circ }{\hbox {C}}$ for 24 h before being used to inoculate the bioreactor at an initial biomass concentration of 0.05 g/L.

Experiments were conducted in Minifors 2 bioreactors (INFORS HT, Switzerland) at a working volume of 1.2 L. The temperature was maintained at 37 °C, and pH was controlled at 6.7 through automated addition of 12.5% ${\hbox {NH}_{4}\hbox {OH}}$. The Dissolved Oxygen Tension (DOT) was maintained at 30% saturation by an agitation (600–1800 RPM) and aeration (0.5$-$2.0 v.v.m) cascade control. The batch phase was continued until complete carbon source depletion, indicated by a sudden decrease in the CER and OUR, accompanied by an increase in DOT. Implementation of CER and OUR soft-sensors was performed according to [51, 52] from the exhaust gas composition measurements of a BlueInOne (Bluesens, Germany) sensor. The subsequent fed-batch phase was initiated equally for all three experiments (Exp. 1, Exp. 2, and Exp. 3) with the feed profile shown in (1).

1 $$\begin{aligned} u_{in}(t) = 0.0022 \cdot \exp [0.17(t-t_{batch})] \quad [\text {L} \,\, \text {h}^{-1}] \end{aligned}$$

Equation (1) considered $u_0=\mu _{set}X_{0}V_0/(Y_{XS}S_{in})$ using a reasonable approximation for the yield $Y_{XS}^\text {guess}=0.5 [g_\text {DCW} \, g^{-1}]=0.65 [\text {c-mol c-mol}^{-1}]$ and with that, the expected biomass concentration ($X_0^\text {guess}=4\,[\text {g L}^{-1}]$) and volume ($V_0=1.2\, [L]$) at the end of the batch can be obtained. The desired growth rate was set to $\mu _{set}=0.17$.

The time $t_{batch}$ represents the time at the end of the batch phase. The feed solution contained glycerol ($S_{in}$) at 750 g/L with all medium components proportionally adjusted to maintain the same molar ratios as in the original DeLisa medium.

### Offline analytics

Offline samples with a volume of 15 mL each were drawn periodically from the reactor and analyzed for biomass and residual substrate concentration. The samples were centrifuged (13400 rpm, 4 $^{\circ }{\hbox {C}}$, 5 min), and supernatants were collected and frozen for later substrate analysis. The pellets were washed with 1 mL ultra-pure water, resuspended for 5 min in the shaker, centrifuged (13400 RPM, 4 $^{\circ }{\hbox {C}}$, 5 min), and dried at 105 $^{\circ }{\hbox {C}}$ for 48 h. The dry cell weight (DCW) content was determined by weighing out the dried pellets. Glycerol and organic acids were quantified using a high-performance liquid chromatography (HPLC) system (Vanquish core, Thermo Scientific, USA) with a ReproGel H column (4.6 x 250 mm, Dr. Maisch, Germany). The column was operated at 70 $^{\circ }{\hbox {C}}$ with 10 mM sulfuric acid as eluent at 0.4 mL/min. The detection was performed with both a refractive index detector (glycerol) and a diode array detector at 210 nm (organic acids). Organic acid production was not detected in these experiments. Both biomass and glycerol measurements were performed in triplicates to determine the central measurement. The analytical dispersion of the biomass measurement considered a ± 5% multiplicative error for biomass values higher than 2.5 g $\hbox {L}^{-1}$ and a fixed ± 0.2 g $\hbox {L}^{-1}$ for lower values. Similarly, our glycerol analytics had a resolution of ± 0.05 g $\hbox {L}^{-1}$.

### Mathematical modeling

Table 2 gives an overview of states, parameters, and constants used in this work.

**Table 2:** Definition of constants, parameters, and state variables

Type	Symbol	Description	Value	Units
Constants	$MW_X$	Biomass molar mass	0.0236	g$_{DCW}$ $\hbox {cmmol}^{-1}$
	$MW_S$	Substrate molar mass	0.0307	g $\hbox {cmmol}^{-1}$
	$m^c$	Maintenance coefficient	1.1	cmmol g$_{DCW}^{-1}$ $\hbox {h}^{-1}$
	$\gamma _X$	Biomass degree of reduction	4.25	–
	$\gamma _{O_2}$	Oxygen degree of reduction	− 4	–
	$\gamma _{S}$	Substrate degree of reduction	4.67	–
	$S_{in}$	Substrate conc. in feed	750	g $\hbox {L}^{-1}$
	$k_{S}$	Substrate saturation constant	0.004	g $\hbox {L}^{-1}$
Parameters	$q_S^{\text {max}}$	Max. glycerol uptake rate	$\ge 0$	g g$_{DCW}^{-1}$ $\hbox {h}^{-1}$
	$Y_{XC}$	Biomass yield on C-source	$\ge 0$	–
TVP$^{\textbf{1}}$	$\lambda$	Substrate uptake adaptability rate	$\ge 0$	g g$_{DCW}^{-1}$ $\hbox {h}^{-2}$
States	*X*	Biomass	$\ge 0$	g$_{DCW}$
	*S*	Substrate	$\ge 0$	g
	$q_S$	Substrate uptake rate	$\ge 0$	g g$_{DCW}^{-1}$ $\hbox {h}^{-1}$
	*V*	Reactor volume	$\ge 0$	L
Inputs	$u_{in}$	Substrate feed	$\ge 0$	L $\hbox {h}^{-1}$
	$F_{out}$	Sampling volume	$\ge 0$	L $\hbox {h}^{-1}$
Outputs	*CER*	${\hbox {CO}_2}$ evolution rate	$\ge 0$	cmmol $\hbox {h}^{-1}$
	*OUR*	${\hbox {O}_2}$ uptake rate	$\le 0$	mmol $\hbox {h}^{-1}$

Time-varying parameter [1]

#### Standard biomass growth model with Monod kinetics

The equations in (2) describe the dynamics of the biomass *X*, substrate *S*, and volume *V* in the reactor. The mechanistic dependence of substrate on $q_S$ and $\mu$ [34] is presented in (3).

2a $$\begin{aligned} \frac{dX}{dt}&= \mu X - F_{\text {out}} X, \end{aligned}$$

2b $$\begin{aligned} \frac{dS}{dt}&= u_{\text {in}} S_{\text {in}} - q_S X - F_{\text {out}} S, \end{aligned}$$

2c $$\begin{aligned} \frac{dV}{dt}&= u_{\text {in}} - F_{\text {out}}. \end{aligned}$$

3a $$\begin{aligned} q_S&= q_S^{\max } \left( \frac{S/V}{k_S + S/V}\right) \end{aligned}$$

3b $$\begin{aligned} \mu&= \left( \frac{q_S}{MW_S} Y_{XC} -m^c\right) MW_X \end{aligned}$$

Although not explicitly considered in Monod’s formulation, the maintenance coefficient $m^c$ accounts for the internal metabolism of the cell and non-growth-associated functions [14, 53, 54].

Finally, the plant output Eq. (4) follow from the biomass stoichiometry, and the assumption of rapid equilibrium in the headspace of the reactor for the CO$_2$ and O$_2$ species [10, 55].

4a $$\begin{aligned} CER&= X \left( \frac{q_S}{MW_S}(1-Y_{X_C}) +m^c \right) \end{aligned}$$

4b $$\begin{aligned} OUR&= \frac{X}{\gamma _{{\hbox {O}_2}}} \left( \frac{q_S}{MW_S}\left( \gamma _XY_{XC}-\gamma _S\right) \right) \end{aligned}$$

#### Dynamic substrate uptake model

To accommodate the evolving and adaptive nature of the microbial system to the exponential phase or changes in the substrate concentration, we propose a model wherein $q_S$ is also a state variable; thus, the system in (2) is extended by (5).

5 $$\begin{aligned} \frac{{dq_{S} }}{{dt}} = \, & \lambda \left( {1 - \frac{{q_{S} }}{{q_{S}^{{{\mathrm{max}}}} }}} \right)\left( {\frac{{S/V}}{{k_{S} + S/V}}} \right) \\ & - q_{S} q_{S}^{{{\mathrm{max}}}} (X/V)\frac{{k_{S} }}{{(k_{S} + S/V)^{2} }} \\ \end{aligned}$$

The negative term in (5) comes from the parametrization of a Monod model for $q_S$ [13] and taking its time derivative in batch mode to preserve the rapid-decaying behavior of $q_S$ near substrate depletion. As for the positive term, the rate of increment of $q_S$ is influenced by the substrate concentration, the time-varying biomass-specific substrate uptake adaptability rate, $\lambda$ [g g$_{DCW}^{-1}$ $\hbox {h}^{-2}$] and a damping term as $q_S$ approaches $q_S^{\max }$.

This formulation explicitly considers $q_S$ to depend on biomass, as suggested in [28], whilst also being a common consideration in bioprocess control at increasing biomass levels [3, 56–58].

The formulation for $\mu$ remains unchanged from Eq. (3b).

### Parameter estimation and parameter confidence interval distribution

Least squares estimation of the parameters $\hat{\boldsymbol{\theta }} = [{\hat{q}}_S^{\max },{\hat{Y}}_{XC}]^T$ was performed using a combination of discontinuous samples, $\boldsymbol{x}_d =[X_d,S_d]^T$, and online measurements $\boldsymbol{y}(t)=[CER(t),OUR(t)]^T$. The unknown parameters were estimated by minimizing the error function *F* (6) using the Nelder-Mead method. All the system states $\boldsymbol{{\hat{x}}}$ and parameters in $\boldsymbol{\hat{\theta }}$ were considered to be non-negative. In this work $k_S$ was assumed known and fixed at 0.004 g $\hbox {L}^{-1}$ [13].

6 $$\begin{aligned} \begin{aligned}&\mathop {\textrm{argmin}}\limits _{\hat{\boldsymbol{\theta }}} \quad F(\boldsymbol{x}_d,\boldsymbol{y};\hat{\boldsymbol{\theta }}) \\&= \sum _k^K \Bigg ( [\boldsymbol{x}_d(t_k) - \hat{\boldsymbol{x}}(t_k, \hat{\boldsymbol{\theta }})]^T \boldsymbol{R} [\boldsymbol{x}_d(t_k) - \hat{\boldsymbol{x}}(t_k, \hat{\boldsymbol{\theta }})] \Bigg ) + \\&\sum _t^T \Bigg ([\boldsymbol{y}(t) - \boldsymbol{h}(\hat{\boldsymbol{x}}(t,\hat{\boldsymbol{\theta }}), \hat{\boldsymbol{\theta }})]^T \boldsymbol{Q} [\boldsymbol{y}(t) - \boldsymbol{h}(\hat{\boldsymbol{x}}(t,\hat{\boldsymbol{\theta }}), \hat{\boldsymbol{\theta }})] \Bigg )\frac{\vert K \vert }{\vert T \vert } \\&\text {subject to:} \\ \quad&\hat{\boldsymbol{\theta }} \ge \boldsymbol{0} \\&\hat{\boldsymbol{x}}(t, \hat{\boldsymbol{\theta }}) \ge \boldsymbol{0}. \\ &\hat{\boldsymbol{x}}(0, \hat{\boldsymbol{\theta }})= \boldsymbol{{x_0}} \\ &\dot{\boldsymbol{x}} = \boldsymbol{f}(\boldsymbol{x},\hat{\boldsymbol{\theta }}) \\ \end{aligned} \end{aligned}$$

The matrices $\boldsymbol{R}$ and $\boldsymbol{Q}$ are diagonal, containing the inverse of the maximum variance recorded for each state and output variable, respectively. A normalization factor $\vert K \vert / \vert T \vert$ was used to compensate for the difference between the number of offline vs online samples. $\boldsymbol{Q}$ was set at 2% $\text {max}_t \;\{CER(t)\}$.

The confidence interval of the estimated parameters $\hat{\boldsymbol{\theta }}$ was approximated empirically via Monte Carlo sampling of random fractions of the dataset (N = 100 per experiment). Each mini-batch contained at least two randomly selected discontinuous samples with analytical noise injected to both biomass and substrate points, and all the online data available between the first and last sample of the mini-batch. The earliest data point in the mini-batch was used for integration of the initial value problem. To prevent parameter overfitting in regions where the objective function was not sensitive to the nominal value of the parameters, the objective function in (6) was minimized using Particle Swarm Optimization (PSO) [59]. The conditions for PSO used in this work were: a swarm size of 100 particles, iterated until the change in the objective function was lower than 0.001 or a maximum of 20 iterations was reached. The minimum step size per particle was also set to 0.001.

### Dynamic $q_S$ model simulation experiment

Consider a system governed by Eqs. (2), (5), growth rate given by (3b) and the system output in (4). For simulation purposes, consider $\lambda (t)$ known and defined by $\lambda (t) = \lambda _{act}(1+\exp (-a(t-t_{act}))^{-1}$ with $\lambda _{act}$: the realized microorganism substrate uptake adaptability rate after the activation time $t_{act}$. Additionally, a simulated input to the system was considered in the form of

7 $$\begin{aligned} u(t) = 0.0014 \cdot \exp \left[ 0.12 \cdot (t - 11.5)\right] \quad [\text {L} \,\text {h}^{-1}] \end{aligned}$$

with $u(t) = 0$ for $t \le 11.5 \,\text {h}$. Equation (7) was formulated in the same manner as Eq. (1), except that the growth rate selected for the simulation experiment was set to $\mu _{set}=0.12 \, \text {h}^{-1}$.

To induce perturbations to the estimated substrate uptake rate $q_S$, Dirac-like spikes were superimposed onto the base feed profile at regular intervals. These spikes were approximated by narrow Gaussian functions centered at $t = \{18, 22, 26, 30\} \,\text {h}$, with a standard deviation of $\sigma = 0.01 \,\text {h}$ and an amplitude proportional to the base signal $2\times u(t)$ at the spike centers.

A simulated plant trajectory was integrated using the following initial conditions and parameters: $X_0$ = 0.05 [g $\hbox {L}^{-1}$], $S_0$ = 8.00 [g $\hbox {L}^{-1}$], $q_S^{0}$ = 0.5 [g g$_{DCW}^{-1}$ $\hbox {h}^{-1}$], $V_0$= 1 [L], $q_S^\text {max}=1.2$ [g g$_{DCW}^{-1}$ $\hbox {h}^{-1}$], $Y_{XC}$ = 0.62 [-], *a*=0.8 [-], $t_{act}$ = 8 [h], $\lambda _{act}=8$ [g g$_{DCW}^{-1}$ $\hbox {h}^{-2}$] using Python’s diffrax Dopri5 solver [60], with an step size of $10^{-6}$ h.

### Particle filter

To estimate the states $\hat{\boldsymbol{x}}=[{\hat{X}},{\hat{S}},{\hat{q}}_S]^T$ and parameters $\boldsymbol{\hat{\theta }}=[{\hat{q}}_S^\text {max},{\hat{Y}}_{XC},\hat{\lambda }]^T$ from the system inputs and outputs, we used a Bayesian sequential Monte Carlo Markov Chain method, also known as a particle filter [41, 61–63].

#### Augmented state-parameter transition

First, consider the parameter-augmented state transition system in Eq. (8).

8 $$\begin{aligned} \begin{bmatrix} d\boldsymbol{{{\hat{x}}}} \\ d\boldsymbol{{\hat{\theta }}} \end{bmatrix} = \begin{bmatrix} \boldsymbol{f}(\boldsymbol{{\hat{x}},\hat{\theta }},\boldsymbol{u}) \\ \boldsymbol{0}_{P,1} \end{bmatrix}dt + \begin{bmatrix} \boldsymbol{0}_{N,1} \\ \boldsymbol{\nu }_{P,1}\end{bmatrix} \end{aligned}$$

With $\boldsymbol{f}(\boldsymbol{{\hat{x}},\hat{\theta }},\boldsymbol{u})$, the system defined by the right hand side of Eqs. (2) and (5), and the mechanistic link given by (3b). $\boldsymbol{0}_{P,1}$ and $\boldsymbol{0}_{N,1}$ are some zero vectors with adequate dimensions for parameters and states, respectively. Additionally, $\boldsymbol{\nu }_{P,1}$ is random noise vector added to $\boldsymbol{\theta }$. In this work, we consider direct Bayesian estimation of $q_S^{\text {max}}$ from the experimentally obtained prior ($\nu _{q_S^\text {max}}=0$), and adaptive estimation for $Y_{XC}$ ($\nu _{Y_{XC}} \sim {\mathcal {N}}(0,10^{-5}\Delta t)$) from the moment $q_S^{\text {max}}$ is found ($\sigma _{q_S}\le 10^{-4}$). $\lambda$ is sampled from a Gamma distribution $\nu _{\lambda } \sim \Gamma (\alpha ,\beta \Delta \phi )$ with $\alpha ,\beta$ some hyperparameters, and $\phi$, an adimensional quantification of cell age. The adaptive noise for the random variable $\lambda$, and the cell age $\phi$ are presented separately in “Adaptive estimation of λ” section.

#### Particle initialization

1000 Particles were sampled from a prior pdf $[\boldsymbol{x}_0,\boldsymbol{\theta }_0] \sim p(\boldsymbol{x}_0,\boldsymbol{\theta }_0)$. *X* and *S* were sampled from a uniform distribution using a spread of 10% from the initial conditions. $Y_{XC}$ and $q_S^\text {max}$ were sampled from the experimental distribution of parameters obtained in “Parameter estimation and parameter confidence interval distribution” section. Given the unknown initial state of $q_S$, each particle was sampled uniformly for $q_S^0$ in the interval $[0,q_S^{\text {max,i}}]$, with $q_S^{\text {max,i}}$ the maximum substrate uptake rate sampled for the particle *i*. Finally, $\lambda$ was initialized at 0 for all particles.

#### Bayesian estimation: likelihood and posterior

At any time $t_{k+1} > t_0$, the transition from $(\boldsymbol{x}_k,\boldsymbol{\theta }_k)$ to $(\boldsymbol{x}_{k+1},\boldsymbol{\theta }_{k+1})$ is given by (8). The posterior distribution $p(\boldsymbol{x}_{1:k},\boldsymbol{\theta }_{1:k} \vert \boldsymbol{y}_{1:k})$ – the probability of observing the state sequence and parameters given a set of observations $\boldsymbol{y}_{1:k}$—is computed using Bayes’ rule as shown in Eq. (9).

9 $$\begin{aligned} p(\boldsymbol{x}_{1:k},\boldsymbol{\theta }_{1:k} \vert \boldsymbol{y}_{1:k}) = \frac{p( \boldsymbol{y}_{1:k} \vert \boldsymbol{x}_{1:k},\boldsymbol{\theta }_{1:k}) p(\boldsymbol{x}_{1:k},\boldsymbol{\theta }_{1:k} \vert \boldsymbol{y}_{1:k-1})}{p(\boldsymbol{y}_{1:k})} \end{aligned}$$

Markov’s property simplifies the information required to compute Bayes’ rule [64, 65]. Therefore, (9) simplifies to (10).

10 $$\begin{aligned} p(\boldsymbol{x}_{k},\boldsymbol{\theta }_{k} \vert \boldsymbol{y}_{1:k}) = \frac{p( \boldsymbol{y}_{k} \vert \boldsymbol{x}_{k},\boldsymbol{\theta }_{k}) p(\boldsymbol{x}_{k},\boldsymbol{\theta }_k \vert \boldsymbol{y}_{1:k-1})}{p(\boldsymbol{y}_{k})} \end{aligned}$$

We aim to construct an estimator whose likelihood function reflects a zero-mean error between the plant output $\boldsymbol{y}(t)$ and the model response $\boldsymbol{h}(\boldsymbol{x},\boldsymbol{\theta })$—as shown in (4)—assuming a defined covariance $\boldsymbol{\Sigma }$ and error dimension *m*, as described in (11) [13, 14].

11 $$\begin{aligned}&p({\textbf{y}}_k\vert \boldsymbol{x}_k,\boldsymbol{\theta }_k) = \frac{1}{2\pi ^{m/2} \det ( \boldsymbol{\Sigma })^{1/2}} \nonumber \\&\exp { \left\{ \frac{1}{2} \left( [\boldsymbol{y}_k - \boldsymbol{h}(\boldsymbol{x_k,\boldsymbol{\theta }_k})]^T \boldsymbol{\Sigma }^{-1} [\boldsymbol{y}_t - \boldsymbol{h}(\boldsymbol{x_k,\boldsymbol{\theta }_k})] \right) \right\} } \end{aligned}$$

The posterior distribution $p(\boldsymbol{\theta }_{k},\boldsymbol{x}_{k} \vert \boldsymbol{y}_{1:k})$ is then approximated through evaluation of the particle ensemble together with the computation of the relative importance ($w_t^i$) for each particle *i* at the time *t* described by (12) [66].

12 $$\begin{aligned} w_k^i = \frac{p({\textbf{y}}_k\vert \boldsymbol{x}^i_k,\boldsymbol{\theta }^i_k) }{\sum _j p({\textbf{y}}_k\vert \boldsymbol{x}^j_k,\boldsymbol{\theta }^j_k)} \end{aligned}$$

Finally, to avoid particle degeneracy,—a common problem in particle filters wherein only a low number of particles have a significant contribution in the posterior distribution [67, 68]—sequential importance resampling (SIR) was implemented whenever the effective number of particles ($N_{eff} = \sum _i w_k^i / \sum _i (w_k^i)^2$) dropped below 50%.

Regarding the covariance matrix $\boldsymbol{\Sigma }$, the scope of this paper only considers biomass as the product of the stoichiometry. Therefore, only the CER dimension was used for estimation to avoid correlation between the residuals ($\boldsymbol{\Sigma }= \sigma ^2_{CER}=0.2,\boldsymbol{y}(t)=y(t)=CER(t)$).

#### Adaptive estimation of $\lambda$

To estimate the time-varying nature of $\lambda$ in Eq. (5), we consider $\lambda \ge 0$, and non-decreasing over time. We propose $\lambda$ to have a Gamma distribution with shape parameter $\alpha$ and scale parameter $\beta$. The reasoning behind using a Gamma process (a stochastic model for the accumulation of effort over a time interval) for $\lambda$ relies on our inability to measure the true adaptability rate of the cells if the error $\boldsymbol{e} = \boldsymbol{y}-\boldsymbol{h}(\boldsymbol{{\hat{x}}},\boldsymbol{{\hat{\theta }}})$ is close to $\boldsymbol{0}$. To solve this, $\hat{\lambda }$ is only resampled from the distribution if the effective number of particles fell below $N_{eff}$, thus creating the event-based condition for the distribution. Therefore, for each particle *i*, the next value of $\hat{\lambda }$ was computed from (13).

13 $$\begin{aligned} \Delta \lambda _i \sim \Gamma (\alpha \Delta \hat{\phi }_i,\beta ) \end{aligned}$$

In this case, $\hat{\phi }$ is defined as the adimensional cell age resulting from integrating $\hat{\mu }(t)$ and maintenance effects from the inoculation to the current fermentation time as shown in (14). Finally, $\Delta \hat{\phi }$ considers the cell aging from the last time $\lambda$ was sampled. In this work a value of $(\alpha ,\beta )=(0.25,1.7)$ was used for the hyperparameters.

14 $$\begin{aligned} \hat{\phi }(t) = \int _{t_0}^{t}\left( \hat{\mu }(\tau ) + {MW_X}\cdot m^c\right) d\tau \end{aligned}$$

#### Kullback–Leibler divergence

The Kullback–Leibler divergence (KLD) [31] in Eq. (15) was employed to quantify the information loss between the experimental distribution of residuals at the step *k* given by $P_k(\boldsymbol{\boldsymbol{e}}) \sim p( \boldsymbol{y}_{k} \vert \boldsymbol{x}_{k},\boldsymbol{\theta }_{k})$ in Eq. (11), and the zero-mean, fixed-variance Gaussian assumption of the estimator $Q(\boldsymbol{\boldsymbol{e}}) \sim {\mathcal {N}}(\boldsymbol{\mu }_q=\boldsymbol{0}, \boldsymbol{\Sigma }_q =\boldsymbol{\Sigma })$.

15 $$\begin{aligned} D_{KL}(P_k \parallel Q) = \int p_k(\boldsymbol{e}) \log \left( \frac{p_k(\boldsymbol{e})}{q(\boldsymbol{e})}\right) d\boldsymbol{e} \end{aligned}$$

Under the assumption that the particles in $P_k$ approximate a Gaussian $P_k \approx {\mathcal {N}}(\boldsymbol{\mu }^k_p, \boldsymbol{\Sigma }^k_p)$, (15) simplifies to the closed-form solution:

16 $$\begin{aligned}&D_{\textrm{KL}}(P_k \parallel Q) = \frac{1}{2} \left[ (\boldsymbol{\mu }_q - \boldsymbol{\mu }^k_p)^\top \boldsymbol{\Sigma }_q^{-1} (\boldsymbol{\mu }_q - \boldsymbol{\mu }^k_p) + \textrm{Tr}\left( \boldsymbol{\Sigma }_q^{-1} \boldsymbol{\Sigma }^k_p\right) \right. \nonumber \\&\left. - \ln \left( \frac{\det (\boldsymbol{\Sigma }^k_p)}{\det (\boldsymbol{\Sigma }_q)}\right) - m \right] \end{aligned}$$

Although the experimental residuals may deviate from a Gaussian distribution, Eq. (16) still provides a sense of distance between the distributions and can be used to compare the performance of the estimator when either the reference Monod model or the dynamic $q_S$ model is used for state and parameter estimation [69, 70].

## Results and discussion

### Least squares parameter estimation reveals systematic bias at the plant output

**Fig. 1:**
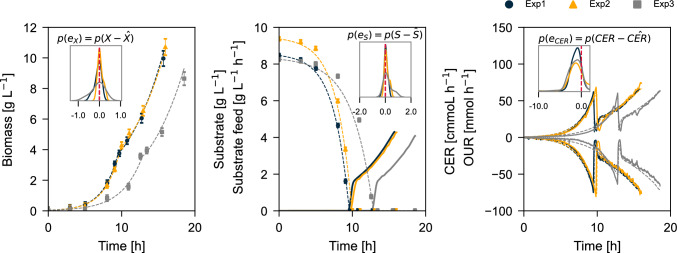
Fermentation profile and parameter estimation for the training setup. Experiments 1, 2, and 3 are biological replicates, with experiment 3 showing a longer lag phase. On each plot, the distribution kernel of the error and its deviation from zero are shown. Solid lines represent plant inputs and outputs, and dashed lines represent the result of the least squares minimization. In the figure on the right, the CER is shown with positive values (generation) and OUR with negative ones (consumption)

To establish whether the original Monod model provides a good representation of the physical process, we performed least squares regression to find the parameters of the standard growth model with Monod kinetics (from now on referred as the Monod model) that minimize the residual error between samples (offline and online) versus the model response. Table 3 shows the parameters obtained for each experiment, and Fig. 1 visually reflects the fit of the data and residuals. It can be seen that Experiments 1 and 2 showed a very similar behavior regarding the duration of the batch phase, which led to similar parameter estimates. Experiment 3, even though it was set up as a biological replicate, showed an extended lag phase, which was compensated by the least squares estimator by assigning a lower $q_S^\text {max}$ (0.81) compared to Experiments 1 (1.05) and 2 (1.04).

**Table 3:** Parameter estimation for the Monod model using nonlinear least squares optimization

Experiment	$q_S^{\max }$ [g $\hbox {gDCW}^{-1}$ $\hbox {h}^{-1}$]	$Y_{XC}$ [-]
Exp. 1	1.05	0.629
Exp. 2	1.04	0.623
Exp. 3	0.81	0.61

These differences in the observed behavior between replicates are a common result in many biological experiments, and although it would be convenient to categorize Experiment 3 as an outlier, it is a plausible realization of an unsupervised experiment at any scale [3, 56]. For all experiments, the error residuals for biomass and substrate are centered at zero. However, a systematic bias is observed in the online signal residuals (Fig. 1) for all cases, suggesting a structural mismatch between the model and the experimental data. For every set of estimated parameters, the model generates CO$_2$ and consumes O$_2$ faster than the plant, questioning whether the parameters found by least squares estimation (${\hat{q}}_S^{\text {max}}$ and ${\hat{Y}}_{XC}$ given known $k_S$) are accurately describing the true microbial parameters and the behavior of the physical plant in study [46, 71].

**Fig. 2:**
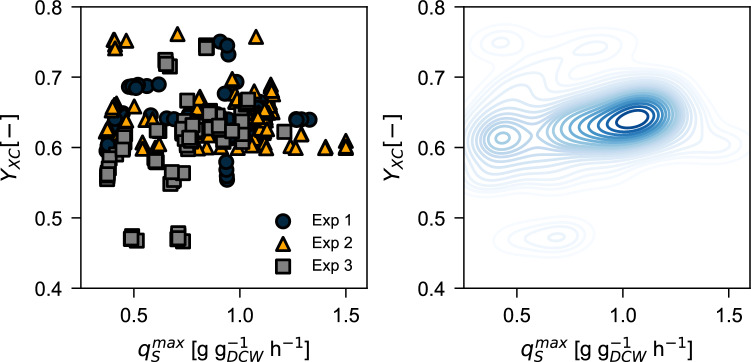
Experimental parameter density distribution from the Monte Carlo Particle Swarm Optimization algorithm. Each experiment was randomly resampled 100 times. The initial conditions for each mini batch were given by the earliest time record in the sample, and at least two offline samples were selected. The PSO bounds for each parameter were $Y_{XC} = [0.3,0.8]$ and $q_S^{\max } = [0.2,1.6]$. The subplot on the right represents the kernel density function $p(\boldsymbol{\theta })$ built from the aggregated results from the left figure

To partially compensate for the systematic bias in the parameter estimation of the Monod model, minimization of the error F (6) was performed using PSO from random fractions of the dataset per experiment as described in “Parameter estimation and parameter confidence interval distribution”. Figure 2 shows the estimation results for 100 randomly sampled mini-batches per experiment from the original dataset, confirming that classical estimation of the Monod parameters is dependent on the initial conditions, number of points, and regime (batch vs carbon-limited phase) [29]. The experimental distribution shows two modes, the global one centered at $q_S^\text {max}=1.06$ [g g$_{DCW}$ $\hbox {h}^{-1}$] and $Y_{XC}=0.63$ and a local cluster at very low $q_S^\text {max}$ values, representing sections that only contained points from the late fed-batch stage, confirming the well-known result that $q_S^\text {max}$ estimation is not possible if the data and signals do not carry any significant information about the parameter [44].

If $\mu _\text {max}$ and/or $q_S^\text {max}$ are only measurable during late stages of the batch phase, an accurate estimation of its value before the carbon-limited phase is crucial for developing robust real-time estimation and control algorithms [3, 4, 13]. Furthermore, if said estimator is to be built from online signals only, any adaptive parameter state and parameter estimation strategy must manage to solve the systematic bias from online signals on early batch stages while maintaining accuracy over the latent variables (biomass and substrate) [5].

### Bayesian estimation of a simulated plant using the dynamic substrate uptake model

**Fig. 3:**
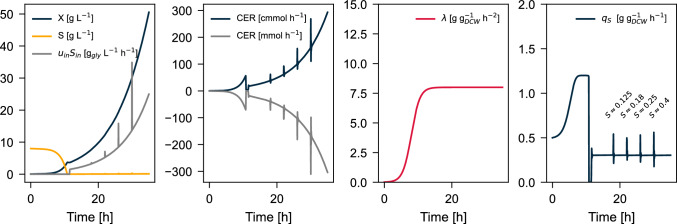
Simulated plant with the dynamic $q_S$ model. The integration step size was $\Delta t=10^{-6}$ h to provide better resolution on the feed-spikes

The time-resolved simulated plant presented in Fig. 3 shows a lagging biological system from inoculation up to $t=8$ hours. $q_S^\text {max}$ Is only reached at later stages of the batch-phase. The objective of this section is to find whether the latent random variable $\lambda$, as well as the system states and parameters, can be estimated only from the inputs and outputs of the plant using Bayesian estimation.

**Fig. 4:**
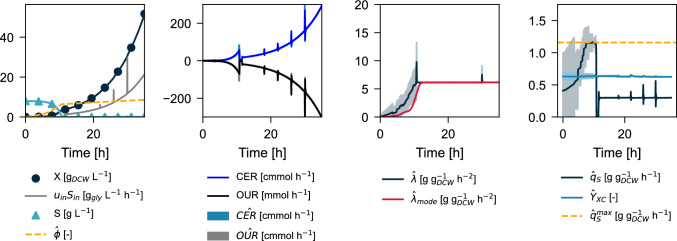
Bayesian estimation for the simulation experiment. A sampling time of $\Delta t = 0.05$ h was chosen. Continuous line represent the current Minimum mean squared error (MMSE) estimates, and the shaded areas correspond to one standard deviation for each state or parameter. Only 10 evenly spaced samples for substrate and biomass are shown for graphical purposes only. OUR is presented with negative values

Figure 4 shows the result of performing particle filtering from the beginning of the fermentation subject to the uncertainty of the initial value for $q_S^{0}$. It can be seen that the cells age faster ($\hat{\phi }$) as the batch reaches the activated phase, which is in accordance with the increasing nature of $q_S$ from $q_S^{0}$ to $q_S^\text {max}$. The estimator slowly discards lower $q_S^\text {max}$ candidates and maintains the upper ones as the model has no evidence whether $q_S^\text {max}$ has been reached unless the effect of $\lambda$ is fully integrated in Eq. (5). As the effective number of particles drops below $N_{eff}$, the event-based condition for the assumed $\Gamma$ distribution of $\lambda$ is triggered, generating for all the resampled particles, new potential substrate uptake adaptability rate trajectories. This type of adaptive resampling is only possible in the PF environment, given the significant non-linearities of (5) , as well as the Gamma distribution assumed for $\lambda$.

Even though the simulated plant reached a $\lambda$ of 8.0 [g g$_{DCW}^{-1}$ $\hbox {h}^{-2}$], the particle filter was unable to identify it (${\hat{\lambda }}=6.1$), presuming that once $q_S^\text {max}$ value is reached, no further information about $\lambda$ can be extracted from the plant. Another important consideration is the difference in sampling times between the simulation ($\Delta t=10^{-6}$ h) and particle filtering ($\Delta t=0.05$ h), which may prevent the confirmation of this adaptability rate on the switch between batch and fed-batch mode.

**Fig. 5:**
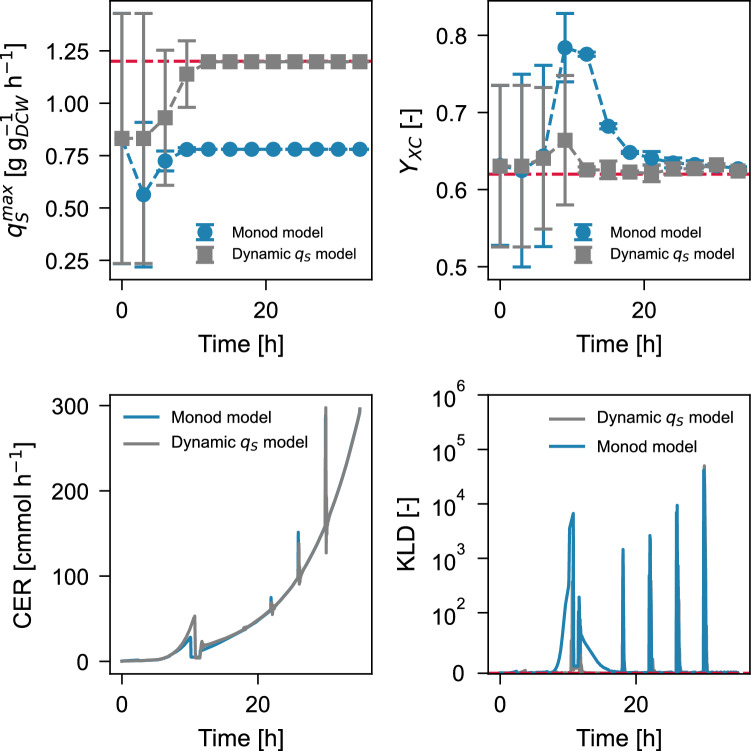
Time evolution for the Bayesian estimation of parameters using the Monod model or the dynamic $q_S$ model as the state transition step. The true parameter values $(q_S^\text {max},Y_{XC})=(1.20,0.62)$ are represented by the dashed lines on each plot. Error bars consider one standard deviation. The Kullback-Leibler divergence between the experimental residual distribution and the estimator design is shown in linear scale up to $10^2$ and in logarithmic scale for higher values

The difference between the true and estimated parameters from Bayesian estimation for the standard Monod and the dynamic $q_S$ model is presented in Fig. 5. Given that the Monod model lacks the degree of freedom provided by the time dependency of $q_S$, a Monod model-based estimator will prematurely discard higher $q_S^\text {max}$ values and would try to compensate for the difference between the output signals and the model response by assuming a higher biomass yield. This compensation, however, stresses the Gaussian likelihood assumption of the particle filter as evidenced in [13].

The bottom right plot of Fig. 5 shows the Kullback–Leibler divergence between the experimental distribution of residuals from particle filtering versus the designed likelihood for the estimator (11). Although the distribution of the residuals is similar for both estimators on early batch and late fed-batch stages, the transition from late-batch to early fed-batch shows a systematic deviation (loss of information in the KLD sense) for the distribution of residuals compared to the reference likelihood function from “Particle filter” section. We show that if a biological system shows significant adaptation dynamics, attempting Bayesian estimation for the states and parameters using a kinetic model for $\mu$ or $q_S$ may lead to the wrong estimates, requiring experimental efforts to localize the augmented state-parameter system, and re-initialization of the filter.

Nevertheless, if the Monod model was to be used for simultaneous estimation of states and parameters, a potential solution to the evidenced problem would be to adaptively estimate both parameters $q_S^\text {max}$ and $Y_{XC}$, but said estimator should also consider the highly correlated nature and relative sensitivity between the parameters during the batch phase [29], requiring a different prior distribution for the parameters than the one shown in Fig. 2.

#### Implications of considering $q_S$ as a dynamic rather than a kinetic expression

Our extension to consider $q_S$ as a state variable has already been phrased in the microbiology literature [25, 26], but a differential equation for its behavior was pending. This new formulation can provide a different interpretation to the pulse experiments from other authors [13, 36, 72].

**Fig. 6:**
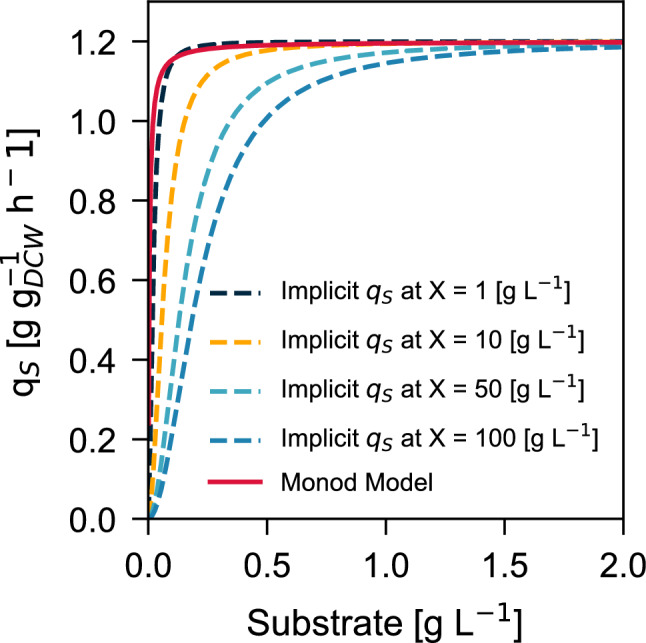
Comparison between steady-state implicit $q_S$ expression and the standard Monod model. The parameters used in this figure were: $q_S^{\text {max}}=1.2$ [g g$_{DCW}^{-1}$ h$^{-1}$], $\lambda =12$ [g g$_{DCW}^{-1}$ h$^{-2}$], $k_S=0.004$ [g L$^{-1}$]

Rather than considering $q_S^\text {max}$ to decay in some way during the carbon-limited phase, Eq. (5) proposes that $q_S$ computation requires, in addition to the substrate levels, information about the current biomass concentration. This allows for an apparent decay of $q_S$ for the same substrate concentration when a steady-state expression is evaluated at different biomass levels (Fig. 6). This implicit, steady-state formulation of $q_S$ can be obtained by setting $dq_S/dt=0$ and solving for $q_S$ as shown in Eq. (17).

17 $$\begin{aligned} q_s(S,X,V) = {\lambda \left( S/V \right) }\left( \frac{q_s^{\text {max}} (X/V) k_s}{k_s + (S/V)} + \frac{\lambda (S/V)}{q_{s}^\text {max}} \right) ^{-1} \end{aligned}$$

Consider the simulated pulse experiments in the carbon-limited phase shown in Fig. 3. It can be seen that even with increasing substrate concentration peaks $S_{peak}=\{0.125,0.18,0.25,0.4\}$ g L$^{-1}$, $q_S^\text {max}$ is not reached, which differs from the simulation experiment by [13], but agrees with the experimental results from the same group and others [72].

### Bayesian estimation of the original dataset using the dynamic $q_S$ model

To validate our proposed model and estimator, we performed Bayesian estimation over the original dataset under the premise that we would be able to determine whether Experiment 3 exhibited a prolonged lag phase, or that the estimated $q_S^\text {max}$ obtained by nonlinear least squares estimation truly reflected the maximum substrate uptake capability of the experiment. Furthermore, analytical uncertainty in the form of a uniform distribution (“Offline analytics” section) is also injected into the initial states of the Particle Filter, reflecting the uncertainty of inoculation and substrate levels.

**Fig. 7:**
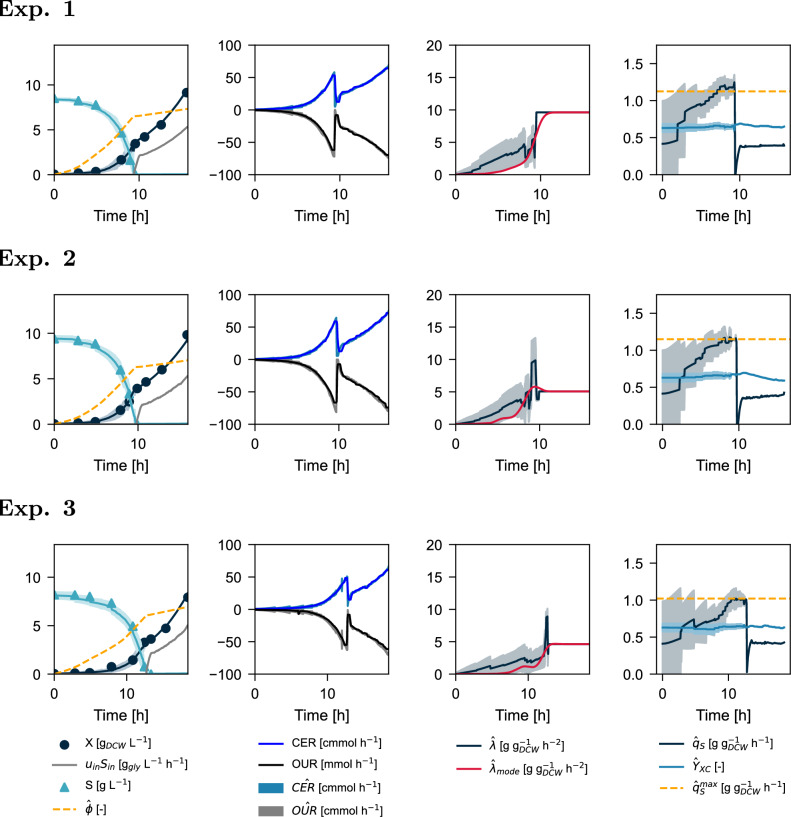
Bayesian estimation of the original dataset using the dynamic $q_S$ model as a step transition model. OUR is presented with negative values

Bayesian estimation of the original dataset is presented in Fig. 7 (from top to bottom: Experiment 1, Experiment 2, and Experiment 3). The model output properly tracked the online signals (CER and OUR), maintaining near-zero residuals throughout the experiment. The correct output tracking in the batch phase significantly improves upon existing state-of-the-art estimation methods in the literature, allowing better approximation of the true value of $q_S^\text {max},Y_{XC}$ while also maintaining plausible biomass and substrate levels compared to the methods in Table 1. In our case, the only required parameter that needs to be fixed is also the least sensitive $k_S$ as already highlighted in [14, 29].

All experiments suggested a dynamic change from an initially unknown $q_s^0$ to $q_s^\text {max}$ during the batch phase. Due to the extra degree of freedom provided by the error-dependent evolution of $\lambda$, better estimates for the states and parameters were possible using Bayesian estimation (Table 4) compared to the least squares method (Table 3). The particle filter estimated $q_S^\text {max}$ to be significantly higher than the values obtained by least-squares estimation (p-value = 0.023), and the yields were comparable.

The extended lag phase of Experiment 3 compared to Experiments 1 and 2 is confirmed by the Maximum a Posteriori (MAP) or mode estimate of the substrate uptake adaptability rate ($\hat{\lambda }_{mode}$). The particle filter maintained a high density of particles with lambdas in the neighborhood of 0 for up to 8 h before activation. In all cases, the MAP profiles for $\lambda$ are similar to the activation functions hypothesized in the literature [25, 73]. Regarding the practical application of our model, the novel parameter $\lambda$ can also be used to capture errors in the initialization of the estimator. If $X_0$ is significantly lower in the plant than the initialized value by the estimator, then $\lambda \approx 0$ until model-plant reconciliation, without discarding $q_S^\text {max}$ or $Y_{XC}$ values. In the opposite case, if $X_0$ is significantly higher in the plant than in the model, $\lambda$ would take higher values. In this scenario, however, the potential true value for $q_S^\text {max}$ could be prematurely discarded if the initialization error is significant.

A noteworthy observation from Fig. 7 occurs at the transition between batch and fed-batch phase for Experiment 3. Even though higher ${\hat{q}}_S^\text {max}$ values were still plausible than the one selected by the estimator at the late batch-phase (Table 4), only those particles that depleted the substrate at the same time as the plant are selected by the sequential importance resampling algorithm. Although there is no Bayesian evidence to consider that the ${\hat{q}}_S^\text {max}$ value obtained by Experiment 3 does not reasonably approximate to the true value, the experimental setup did not allow further testing for potentially higher values, as it would have required a slightly more prolonged batch phase, and consideration of heteroskedasticity in this region to expand the variance of the residuals [74]. Beyond the consideration of experimental conditions, Bayesian estimation from these nonlinear models in bioprocess engineering could be further improved by incorporating a KLD-based compensation metric when attempting particle resampling [75] and by properly addressing the estimator bounds [76].

**Table 4:** Real-time parameter estimation from the model-observer approach

Parameter	$Y_{XC}(t_f)$ [-]	$q_S^{\text {max}}$ [g g$_{DCW}^{-1}$ h $^{-1}$]
Exp. 1	0.63	1.17
Exp. 2	0.61	1.18
Exp. 3	0.62	1.05

#### Discussion on model and estimator selection

The parameters, states, and output trajectory obtained by Bayesian estimation differ significantly from the ones obtained by least squares (Table 3 and Fig. 1). Although least squares estimation can be used to retrieve an experimental distribution for the parameters (Fig. 2), it is important to acknowledge that if real-time estimation is to be performed from online signals such as the plant outputs, then an evaluation of the maximum-likelihood estimator assumptions: zero-mean residuals, homoscedasticity and uncorrelated errors over the online signals versus the model output is required. Otherwise, the methodologies suggested in the literature regarding the parameter and state confidence intervals may not apply [23, 46, 49].

However, Bayesian estimation by itself is not sufficient to improve the accuracy of the estimated parameters and states. For instance, Müller et al., 2023 [13], attempted Bayesian estimation while simultaneously estimating $q_S^\text {max}$ and $Y_{XC}$ under the assumption of $q_S$ being a kinetic rather than a dynamic. However, their results presented a significant plant-model mismatch in early stages, leading to compromised accuracy of their estimator. We quantified this drift using the Kullback–Leibler divergence between the Monod model and the dynamic $q_S$ model in Fig. 5 and allowed us to hypothesize the structural change required in the form of Eq. (5).

Given our inability to mechanistically and effectively model the adaptation phase of microorganisms to changes in substrate concentrations, the consideration of $\lambda$ as a random variable is justified. Although the selection of hyperparameters $\alpha$ and $\beta$ was not discussed, algorithms like the Expectation Maximization [77] could be implemented over a larger dataset to gain insights into a potential prior for $\alpha$ and $\beta$.

The choice to model $q_S$ as a state variable rather than a kinetic expression, and the incorporation of the random variable $\lambda$, allows for a better approximation of the entire system to approach a maximum likelihood estimator. The insights gained from this work can give additional information for model adaptive and predictive control sections strategies [3], particularly regarding the maximum rate of change for input signals ($\Delta u_k(\lambda )$), to maintain the system culture at $q_S(t)=q_S^\text {set}(t)$ and prevent undesired accumulation of substrate, which may activate overflow metabolism under stressful conditions.

Finally, our dynamic $q_S$ expression in Eq. (5) provides an integrative solution to the decaying $q_S^\text {max}$ behavior required by Monod-like models in estimation and control environments [3]. As biomass accumulates inside the reactor, a higher substrate concentration is needed to maintain the same gradient. This effect is highlighted in Fig. 6. If (5) can be used to provide a reasonable explanation to the pulse experiments [36], as well as integrating the experimental observations from Krichen et al. [28], then the focus of adaptive state and parameter estimation can be set on the changes of $Y_{XC}$, particularly during induction or production stages, wherein the information contained in *OUR*(*t*) is no longer correlated with *CER*(*t*), allowing for the design of more robust estimators in the future.

## Conclusion

This contribution aimed to design a robust real-time model-observer that, in addition to providing reliable state and parameter estimates, accounts for the dynamics of microbial adaptation directly into its formulation. To the best of our knowledge, this is the first publication to explicitly consider the substrate uptake rate as a state variable, and Eq. (5) to be a plausible differential formulation.

The incorporation of $q_S$ as a state variable improves the performance of the particle filter, especially in early-batch stages wherein the assumption of $q_S=q_S^\text {max}$ of the Monod model may not hold. State-of-the-art estimation methods from online measurements in bioprocess engineering are now further improved by the novel feature of our estimator: the determination, in real-time, of the substrate uptake adaptability rate of the system. Compared to the literature survey from Table 1, our estimator matches the requirements from [13], requiring only $k_S$ to perform the reconstruction of the system from online signals, but allow simultaneous estimation of state and parameters, from properly defined prior probabilities, extending the practical implementation of the estimator.

The acknowledgment of the random variable $\lambda$, and the biomass dependency of $q_S$ allows a different interpretation of the pulse experiment observations from other groups. We argue that estimation of $q_S^\text {max}$ or $\mu _\text {max}$ using the Monod model as a reference may be difficult in both batch and carbon-limited phases due to the influence of the time-scale effect of microbial adaptability.

Our model-observer framework can be extended to consider multiple substrates and gain better insights into pathway regulation and overflow metabolism. For instance, it is a known effect that high-cell-density fed-batch fermentations tend to accumulate acetate or ethanol even if $q_S<< q_S^\text {max}$. A mixture of effects between oxygen uptake rate and the substrate gradient proposed by the $q_S$ balance equation may allow the formulation of better hypotheses about the relationship between $S,q_S, X$, and side products. Also, similar approaches to the activation of $\lambda$ could be implemented to evaluate the hypothesis of acetate overflow metabolism, as the transition mechanism of *E. coli* concerning acetate formation is often sharp [78]. Nevertheless, our estimator presents a significant improvement over current estimation methods and allows the development and implementation of better robust real-time control algorithms in the bioprocess engineering field.

## Data Availability

Data and algorithms are available from the corresponding author upon reasonable request.
